# Validation of Abstract Side-Channel Models for Computer Architectures

**DOI:** 10.1007/978-3-030-53288-8_12

**Published:** 2020-06-13

**Authors:** Hamed Nemati, Pablo Buiras, Andreas Lindner, Roberto Guanciale, Swen Jacobs

**Affiliations:** 8 Microsoft Research Lab, Redmond, WA USA; 9 University of Southern California, Los Angeles, CA USA; 10 Helmholtz Center for Information Security (CISPA), Saarbrücken, Germany; 11 KTH Royal Institute of Technology, Stockholm, Sweden

**Keywords:** Testing, Side channels, Information flow security, Model validation, Microarchitectures

## Abstract

Observational models make tractable the analysis of information flow properties by providing an abstraction of side channels. We introduce a methodology and a tool, Scam-V, to validate observational models for modern computer architectures. We combine symbolic execution, relational analysis, and different program generation techniques to generate experiments and validate the models. An experiment consists of a randomly generated program together with two inputs that are observationally equivalent according to the model under the test. Validation is done by checking indistinguishability of the two inputs on real hardware by executing the program and analyzing the side channel. We have evaluated our framework by validating models that abstract the data-cache side channel of a Raspberry Pi 3 board with a processor implementing the ARMv8-A architecture. Our results show that Scam-V can identify bugs in the implementation of the models and generate test programs which invalidate the models due to hidden microarchitectural behavior.

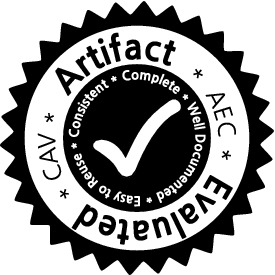



## Introduction

Information flow analysis that takes into account side channels is a topic of increasing relevance, as attacks that compromise confidentiality via different microarchitectural features and sophisticated side channels continue to emerge [2, 27, 28, 31–33, 40]. While there are information flow analyses that try to counter these threats [3, 15], these approaches use models that abstract from many features of modern processors, like caches and pipelining, and their effects on channels that can be accessed by an attacker, like execution time and power consumption. Instead, these models [36] include explicit “observations” that become available to an attacker when the program is executed and that should overapproximate the information that can be observed on the real system.

While abstract models are indispensable for automatic verification because of the complexity of modern microarchitectures, the amount of details hidden by these models makes it hard to trust that no information flow is missed, i.e., their soundness. Different implementations of the same architecture, as well as optimizations such as parallel and speculative execution, can introduce side channels that may be overlooked by the abstract models. This has been demonstrated by the recent Spectre attacks [32]: disregarding these microarchitectural features can lead to consider programs that leak information on modern CPUs as secure. Thus, it is essential to validate whether an abstract model adequately reflects all information flows introduced by the low-level features of a specific processor.

In this work, we introduce an approach that addresses this problem: we show how to validate observational models by comparing their outputs against the behavior of the real hardware in systematically generated experiments. In the following, we give an overview of our approach and this paper.

**Fig. 1.**
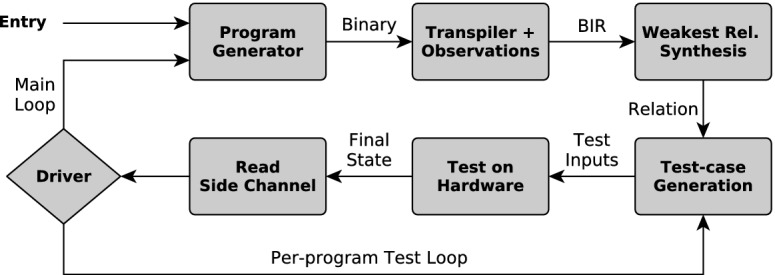
Validation framework workflow

**Our Contribution.** We introduce Scam-V (Side Channel Abstract Model Validator), a framework for the automatic validation of abstract observational models. At a high level, Scam-V generates well-formed^1^ random binaries and attempts to construct pairs of initial states such that runs of the binaries from these states are indistinguishable at the level of the model, but distinguishable on the real hardware. In essence, finding such counterexamples implies that the observational model is not sound, and leads to a potential vulnerability. Figure 1 illustrates the main workflow of Scam-V.

The first step of our workflow (described in Sect. 3) is the generation of a binary program for the given architecture, guided towards programs that trigger certain features of the architecture. The second step translates the program to the intermediate language BIR (described in Sect. 2.4) and annotates the result with observations according to the observational model under validation. This transpilation is provably correct with respect to the formal model of the ISA, i.e., the original binary program and the transpiled BIR program have the same effects on registers and memory. In step three we use symbolic execution to synthesize the weakest relation on program states that guarantees indistinguishability in the observational model (Sect. 4). Through this relation, the observational model is used to drive the generation of *test cases* – pairs of states that satisfy the relation and can be used as inputs to the program (Sect. 5). Finally, we run the generated binary with different test cases on the real hardware, and compare the measurements on the side channel of the real processor. A description of this process together with general remarks on our framework implementation are in Sect. 6. Since the generated test cases satisfy the synthesized relation, soundness of the model would imply that the side-channel data on the real hardware cannot be distinguished either. Thus, a test case where we can distinguish the two runs on the hardware amounts to a counterexample that invalidates the observational model. After examining a given test case, the driver of the framework decides whether to generate more test cases for the same program, or to generate a new program.

We have implemented Scam-V in the HOL4 theorem prover^2^ and have evaluated the framework on three observational models (introduced in Sect. 2.3) for the L1 data-cache of the ARMv8 processor on the Raspberry Pi 3 (Sect. 2.2). Our experiments (Sect. 7) led to the identification of model invalidating microarchitectural features as well as bugs in the ARMv8 ISA model and our observational extensions. This shows that many existing abstractions are substantially unsound.

Since our goal is to validate that observational models overapproximate hardware information flows, we do not attempt to identify practically exploitable vulnerabilities. Instead, our experiments attempt to validate these models in the worst case scenario for the victim. This consists of an attacker that can precisely identify the cache lines that have been evicted by the victim and that can minimize the noise of these measurements in the presence of background processes and interrupts.

## Background

### Observational Models

We briefly introduce the concepts of side channels, indistinguishability, observational models, and observational equivalence. For the rest of this section, consider a fixed program that runs on a fixed processor. We can model the program running on the processor by a transition system $M=\left\langle S,\rightarrow \right\rangle$, where $S$ is a set of states and $\rightarrow \subseteq S\times S$ a transition relation. In automated verification, the state space of such a model usually reflects the possible values of program variables (or: registers of the processor), abstracting from low-level behavior of the processor, such as cache contents, electric currents, or real-time behavior. That is, for every state of the real system there is a state in the model that represents it, and a state of the model usually represents a set of states of the real system.

Then, a *side channel* is a trait of the real system that can be read from by an attacker and that is not modeled in *M*.

#### Definition 1

**(Indistinguishability).** States $r_1$ and $r_2$ of the real system are *indistinguishable* if a real-world attacker is not able to distinguish executions from $r_1$ or $r_2$ by means of the side channel on the real hardware.

Note that executions may be distinguishable even if they end in the same final state, e.g., if the attacker is able to measure execution time.

In order to verify resilience against attacks that use side channels, one option is to extend the model to include additional features of the real system and to formalize indistinguishability in terms of some variations of non-interference [25, 26]. Unfortunately, it is infeasible to develop formal models that capture *all* side channels of a modern computer architecture. For instance, precisely determining execution time or power consumption of a program requires to deal with complex processor features such as cache hierarchies, cache replacement policies, speculative execution, branch prediction, or bus arbitration. Moreover, for some important parts of microarchitectures, their exact behavior may not even be public knowledge, e.g., the mechanism used to train the branch predictor. Additionally, information flow analyses cannot use the same types of overapproximations that are used for checking safety properties or analyzing worst-case execution time, e.g., the introduction of nondeterminism to cover all possible outcomes.

In order to handle this complexity, information flow analyses [3, 15] use models designed to overapproximate information flow to channels in terms of system state observations. To this end, the model is extended with a set of possible observations *O* and we consider a transition relation $\rightarrow \subseteq S\times O \times S$, i.e., each transition produces an observation that captures the information that it potentially leaks to the attacker. We assume that the set *O* contains an *empty observation* $\perp$, and call a transition labeled with $\perp$ a *silent* transition. We call the resulting transition system an *observational model*. For instance, in case of a rudimentary cacheless processor, the execution time of a program depends only on the sequence of executed instructions. In this case, extending the model with observations that reveal the instructions is more convenient than producing a clock-accurate model of the system.

We use the operator $\circ$ for the sequential composition of observations. In particular, for a trace $\pi = s_0 \rightarrow ^{o_1} s_1 \dots \rightarrow ^{o_n} s_n$ of the model, we write $o_1 \circ \dots \circ o_n$ for the sequence of observations along $\pi$. We write $o_1 \circ \dots \circ o_n \approx o_1' \circ \dots \circ o_{n'}'$ if the two sequences are equal after removing silent transitions. Comparing traces with observations leads to a notion of *observational equivalence*, defined as a relation on program states.

#### Definition 2

**(Observational equivalence).** Traces $\pi = s_0 \rightarrow ^{o_1} s_1 \dots \rightarrow ^{o_n} s_n$ and $\pi ' = s'_0 \rightarrow ^{o'_1} s'_1 \dots \rightarrow ^{o'_{n'}} s'_{n'}$ of an observational model *M* are *observationally equivalent* (written as $\pi \sim _M \pi '$) iff $o_1 \circ \dots \circ o_n \approx o'_1 \circ \dots \circ o'_{n'}$.

States $s_1 \in S$ and $s_2 \in S$ are *observationally equivalent*, denoted $s_1 \sim _M s_2$, iff for every possible trace $\pi _1$ of *M* that starts in $s_1$ there is a trace $\pi _2$ of *M* that starts in $s_2$ such that $\pi _1 \sim _M \pi _2$, and vice versa.

Note that this notion is, in principle, different from the notion of *indistinguishability*. The overapproximation of information flows can lead to false positives: for example, execution of a program may require the same amount of time even if the sequences of executed instructions are different. A more severe concern is that these abstractions may overlook some flows of information due to the number of low-level details that are hidden. For instance, an observational model may not take into account that for some microcontrollers the number of clock cycles required for multiplication depends on the value of the operands.

The use of an abstract model to verify resilience against side-channel attacks relies on the assumption that observational equivalence entails indistinguishability for a real-world attacker on the real system:

#### Definition 3

**(Soundness).** An observational model *M* is *sound* if whenever the model states $s_1$ and $s_2$ represent the real system states $r_1$ and $r_2$, respectively, then $s_1 \sim _M s_2$ entails indistinguishability of $r_1$ and $r_2$.

### The Evaluation Platform: Raspberry Pi 3

In order to evaluate our framework, we selected Raspberry Pi 3^3^, which is a widely available ARMv8 embedded system. The platform’s CPU is a Cortex-A53, which is an 8-stage pipelined processor with a 2-way superscalar and in-order execution pipeline. The CPU implements branch prediction, but it does not support speculative execution. This makes the CPU resilient against variations of Spectre attacks [5].

In the following, we focus on side channels that exploit the Level 1 (L1) data-cache of the system. The L1 data-cache is transparent for programmers. When the CPU needs to read a location in memory in case of a cache miss, it copies the data from memory into the cache for subsequent uses, tagging it with the memory location from which the data was read.

**Fig. 2.**
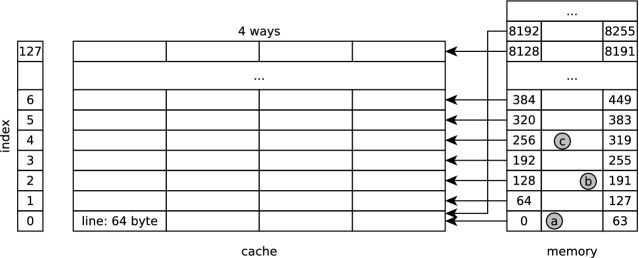
L1 data-cache structure.

Data is transferred between memory and cache in blocks of 64 bytes, called cache lines. The L1 data-cache (Fig. 2) is physically indexed and physically tagged and is 4-way set associative: each memory location can be cached in four different entries in the cache—when a line is loaded, if all corresponding entries are occupied, the CPU uses a specific (and usually underspecified) replacement policy to decide which colliding line should be evicted. The whole L1 cache is 32KB in size, hence it has 128 cache sets (i.e. 32 KB/64 B/4). Let *a* be a physical address, in the following we use $\texttt {off}(a)$ (i.e., least significant 6 bits), $\texttt {index}(a)$ (i.e., bits from 6 to 12), and $\texttt {tag}(a)$ (i.e., the remaining bits) to extract the cache offset, cache set index, and cache tag of the address.

The cache implements a prefetcher, for some configurable $k \in \mathbb {N}$: when it detects a sequence of *k* cache misses whose cache set indices are separated by a fixed stride, the prefetcher starts to fetch data in the background. For example, in Fig. 2, if $k=3$ and the cache is initially empty then accessing addresses *a*, *b*, and *c*, whose cache lines are separated by a stride of 2, can cause the cache to prefetch the block $[384 \dots 449]$.

### Different Attacker and Observational Models

Attacks that exploit the L1 data-cache are usually classified in three categories: In *time-driven attacks* (e.g. [47]), the attacker measures the execution time of the victim and uses this knowledge to estimate the number of cache misses and hits of the victim; In *trace-driven attacks* (e.g. [1, 48]), the adversary can profile the cache activities during the execution of the victim and observe the cache effects of a particular operation performed by the victim; Finally, in *access-driven attacks* (e.g. [39, 46]), the attacker can only determine the cache sets modified after the execution of the victim has completed. A widely used approach to extract information via cache is Prime+Probe [40]: (1) the attacker reads its own memory, filling the cache with its data; (2) the victim is executed; (3) the attacker measures the time needed to access the data loaded at step (1): slow access means that the corresponding cache line has been evicted in step (2).

In the following we disregard time-driven attacks and trace-driven attacks: the former can be countered by normalizing the victim execution time; the latter can be countered by preventing victim preemption. Focusing on access-driven attacks leads to the following notion of indistinguishability:

#### Definition 4

Real system states $r_1$ and $r_2$ are *indistinguishable for access-driven attacks on the L1 data-cache* iff executions starting in $r_1$ or $r_2$ modify the same cache sets.

We remark that for multi-way caches, the need for models that overapproximate the information flow is critical since the replacement policies are seldom formally specified and a precise model of the channel is not possible. The following observational model attempts to overapproximate information flows for data-caches by relying on the fact that accessing two different addresses that only differ in their cache offset produces the same cache effects:

#### Definition 5

The transition relation of the *multi-way cache and pc observational model* is $$s\rightarrow _{mwc, pc}^o s'$$, where $$\rightarrow _{mwc,pc}^o$$ models the execution of one single instruction, with $$o \in \mathbb {N}\times ((\{rd,wt\} \times \mathbb {N}\times \mathbb {N})\ \cup \perp )$$. If $$o=(pc, acc )$$ then *pc* is the current program counter and $$ acc =( op ,t,i)$$ is the memory access performed by the instruction, where $$ op $$ is the memory operation, $$t$$ is the cache tag and $$i$$ is the cache set index corresponding to the address. If the instruction does not access the memory, then $$ acc =\perp $$.

Notice that by making the program counter observable, this model assumes that the attacker can infer the sequence of instructions executed by the program.

We introduce several relaxed models, representing different assumptions on the hardware behavior and attacker capability. Each relaxed model is obtained by projecting observations of Definition 5. Let $\alpha$ be a relaxed model and $f_{\alpha }$ the corresponding projection function, then $s\rightarrow _{\alpha }^{o'} s'$ iff exists *o* such that $f_{\alpha }(o) = o'$ and $s\rightarrow _{mwc, pc}^o s'$.

The following model assumes that the effects of instructions that do not interact with the data memory are not measurable, hence the attacker does not observe the program counter:

#### Definition 6

The projection of the *multi-way cache observational model* is $$f_{mwc}((pc, acc )) = acc $$.

On many processors, the replacement policy for a cache set does not depend on previous accesses performed to other cache sets. The resulting isolation among cache sets leads to the development of an efficient countermeasure against access-driven attacks: cache coloring [23, 45]. This consists in partitioning the cache sets into multiple regions and ensuring that memory pages accessible by the adversary are mapped to a specific region of the cache. In this case, accesses to other regions do not affect the state of cache sets that an attacker can examine. Therefore these accesses are not observable. This assumption is captured by the following model:

#### Definition 7

The projection of the *partitioned multi-way cache observational model* is $$f_{pmwc}((pc, acc )) = acc $$ if $$ acc = ( op , t, i)$$ and $$i$$ belongs to the set of cache sets that are addressable by the attacker, and is $$\perp $$ otherwise.

Notice that cache prefetching can violate soundness of this model, since accesses to the non-observable region of the cache may lead to prefetching addresses that lie in the observable part of the cache (see Sect. 7.2).

Finally, for direct-mapped caches, where each memory address is mapped to only one cache entry, the cache tag should not be observable if the attacker does not share memory with the victim:

#### Definition 8

The projection of the *direct-mapped cache observational model* is $f_{dc}((pc, ( op ,t,i))) = ( op ,i)$ and $f_{dc}((pc, \perp )) = \perp$.

Since the cache in Cortex-A53 is multi-way set associative, this model is not sound. For example, in a two-way set associative cache, accessing *a*, *a* and *a*, *b*, where both *a* and *b* have the same cache set index but different cache tags, may result in different cache states.

**Fig. 3.**
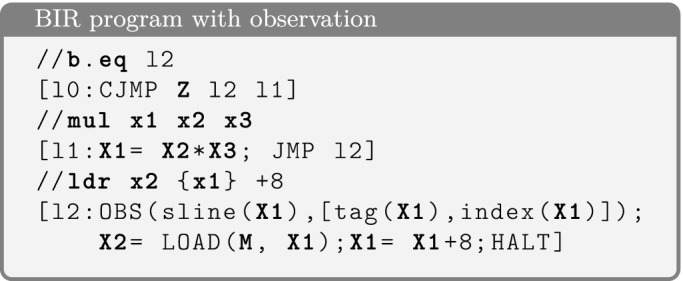
BIR transpilation example

### Binary Intermediate Representation

To achieve a degree of hardware independence, we use the architecture-agnostic intermediate representation BIR [34]. It is an abstract assembly language with statements that work on memory, arithmetic expressions, and jumps. Figure 3 shows an example of code in a generic assembly language and its transpiled BIR code. This code performs a conditional jump to *l2* if **Z** holds, and otherwise it sets $\mathbf {X1}$ to the multiplication $\mathbf {X2 * X3}$. Then, at *l2* it loads a word from memory at address $\mathbf {X1}$ into $\mathbf {X2}$, and finally adds 8 to the pointer $\mathbf {X1}$. BIR programs are organized into blocks, which consist of jump-free statements and end in either conditional jump (CJMP), unconditional jump (JMP), or HALT.

BIR also has explicit support for *observations*, which are produced by statements that evaluate a list of expressions in the current state. To account for expressive observational models, BIR allows conditional observation. The condition is represented by an expression attached to the observation statement. The observation itself happens only if this condition evaluates as true in the current state. The observations in Fig. 3 reflect a scenario where the data-cache has been partitioned: some lines are exclusively accessible by the victim (i.e. the program), some lines can be shared with the attacker. The statement OBS(sline($\mathbf {X1}$), [tag($\mathbf {X1}$), index($\mathbf {X1}$)]) for the load instruction consists of an observation condition (sline($\mathbf {X1}$)) and a list of expressions to observe ([tag($\mathbf {X1}$), index($\mathbf {X1}$)]). The function sline checks that the argument address is mapped in a shared line and therefore visible to the attacker. The functions tag and index extract the cache tag and set index in which the argument address is mapped. Binary programs can be translated to BIR via a process called *transpilation*. This transformation reuses formal models of the ISAs and generates a proof that certifies correctness of the translation by establishing a bisimulation between the two programs.

## Program Generation

We base our validation of observational models on the execution of binary programs rather than higher-level code representations. This approach has the following benefits: (i) It obviates the necessity to trust compilers or reason about how their compilation affects side-channels. (ii) Implementation effort is reduced because most existing side-channel analysis approaches also operate on binary representations, which requires ISA models. (iii) This approach allows to find ISA model faults independently of the compilation. (iv) It enables a unified infrastructure to handle many different types of channels.

**Fig. 4.**
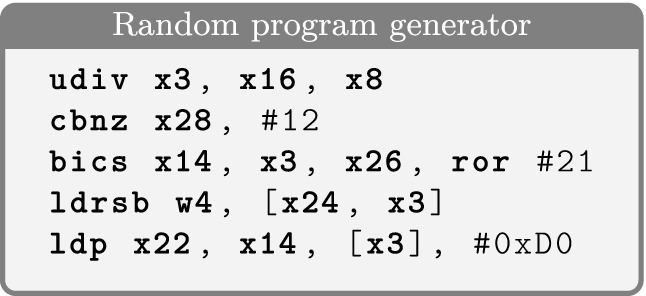
Example programs generated by the Scam-V random program generator.

**Fig. 5.**
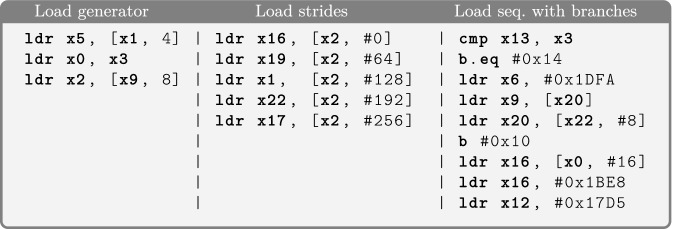
Example programs generated by Scam-V monadic program generators.

In Scam-V, we implemented two techniques to generate well-formed binaries: *random* program generation and *monadic* program generation. The random generator leverages the instruction encoding machinery from the existing HOL4 model of the ISA and produces arbitrary well-formed ARMv8 binaries, with the possibility to control the frequency of occurrences of each instruction class. The monadic generator is following a grammar-driven approach in the style of QuickCheck [13] that generates arbitrary programs that fit a specific pattern or template. The program templates can be defined in a modular, declarative style and are extensible. We use this approach to generate programs in a guided fashion, focusing on processor features that we want to exercise in order to validate a model, or those we suspect may lead to a counterexample. Figures 4 and 5 show some example programs generated by Scam-V, including straight-line programs that only do memory loads, programs that load from addresses in a stride pattern to trigger automatic prefetching, and programs with branches. More details on how the program generators work can be found in [38].

## Synthesis of Weakest Relation

Synthesis of the weakest relation is based on standard symbolic execution techniques. We only cover the basic ideas of symbolic execution in the following and refer the reader to [30] for more details. We use $\mathbf {X}$ to range over symbols, and $\mathbf {c}$, $\mathbf {e}$, and $\mathbf {p}$ to range over symbolic expressions. A symbolic state $\sigma$ consists of a concrete program counter $i_{\sigma }$, a path condition $\mathbf {p}_{\sigma }$, and a mapping $\mathbf {m}_{\sigma }$ from variables to symbolic expressions. We write $e(\sigma ) = \mathbf {e}$ for the symbolic evaluation of the expression *e* in $\sigma$, and $\mathbf {e}(s)$ for the value obtained by substituting the symbols of the symbolic expression $\mathbf {e}$ with the values of the variables in *s*, where *s* is a concrete state.

Symbolic execution produces one terminating state^4^ for each possible execution path: a terminating state is produced when $\texttt {HALT}$ is encountered; the execution of $\texttt {CJMP}\ c\ l_1\ l_2$ from state $\sigma$ follows both branches using the path conditions $c(\sigma )$ and $\lnot c(\sigma )$. Symbolic execution of the example in Fig. 3 produces the terminating states $\sigma _1$ and $\sigma _2$. For the first branch we have $\mathbf {p}_{\sigma _1} = \mathbf {Z}$ and $\mathbf {m}_{\sigma _1} = \{ {X_1} \rightarrow {\mathbf {X}_1+8}, {X_2} \rightarrow {\texttt {LOAD}(\mathbf {M}, \mathbf {X}_1)} \}$ (we omit the variables that are not updated), and for the second branch $\mathbf {p}_{\sigma _2} = \lnot \mathbf {Z}$ and $\mathbf {m}_{\sigma _2} = \{ {X_1} \rightarrow {\mathbf {X}_2 * \mathbf {X}_3 + 8}, {X_2} \rightarrow {\texttt {LOAD}(\mathbf {M}, \mathbf {X}_2 * \mathbf {X}_3)} \}$.

We extend standard symbolic execution to handle observations. That is, we add to each symbolic state a list $\mathbf {l}_{\sigma }$, and the execution of  in $\sigma$ appends the pair  to $\mathbf {l}_{\sigma }$, where $\mathbf {c} = c(\sigma )$ and  are the symbolic evaluation of the condition and expressions of the observation. For instance, in the example of Fig. 3 the list for the terminating states are

$$ \begin{array}{ll} \mathbf {l}_{\sigma _1} &{} = [(\texttt {sline}(\mathbf {X}_1), [\texttt {tag}(\mathbf {X}_1), \texttt {index}(\mathbf {X}_1)])]\\ \mathbf {l}_{\sigma _2} &{} = [ (\texttt {sline}(\mathbf {X}_2 *\mathbf {X}_3), [\texttt {tag}(\mathbf {X}_2 * \mathbf {X}_3), \texttt {index}(\mathbf {X}_2 * \mathbf {X}_3)])] \end{array} $$

Let $\Sigma$ be the set of terminating states produced by the symbolic execution, *s* be a concrete state, and $\sigma \in \Sigma$ be a symbolic state such that $\mathbf {p}_{\sigma }(s)$ holds, then executing the program from the initial state *s* produces the value $\mathbf {m}_{\sigma }(X)(s)$ for the variable *X*. Moreover, let , then the generated observations are , where  (i.e. observations are list of concrete values).

After computing $\Sigma$, we synthesize the observational equivalence relation (denoted by $\sim$) by ensuring that every possible pair of execution paths have equivalent lists of observations. Formally, $s_1 \sim s_2$ is equivalent to:

$$ \bigwedge _{(\sigma _1, \sigma _2) \in \Sigma \times \Sigma } (\mathbf {p}_{\sigma _1}(s_1) \wedge \mathbf {p}_{\sigma _2}(s_2) \Rightarrow \mathbf {l}_{\sigma _1}(s_1) = \mathbf {l}_{\sigma _2}(s_2)) $$

This synthesized relation implies the observational equivalence defined in Sect. 2 (Definition 2). In the example, the synthesized relation (after simplification) is as follows (notice that primed symbols represent variables of the second state and we omitted the symmetric cases):

$$\begin{aligned} \begin{array}{ll} (\mathbf {Z}\; \wedge \; \mathbf {Z'})\; \Rightarrow \\ \;\;\left( \begin{array}{l} \texttt {sline}(\mathbf {X}_1) = \texttt {sline}(\mathbf {X'}_1)\; \wedge \\ \texttt {sline}(\mathbf {X}_1) \Rightarrow (\texttt {tag}(\mathbf {X}_1) = \texttt {tag}(\mathbf {X'}_1) \wedge \texttt {index}(\mathbf {X}_1) = \texttt {index}(\mathbf {X'}_1))\\ \end{array}\right) \;\; \wedge \\ (\mathbf {Z} \wedge \lnot \mathbf {Z'}) \Rightarrow \\ \;\;\left( \begin{array}{l} \texttt {sline}(\mathbf {X}_1) = \texttt {sline}(\mathbf {X'}_2 * \mathbf {X'}_3)\; \wedge \\ \texttt {sline}(\mathbf {X}_1) \Rightarrow (\texttt {tag}(\mathbf {X}_1) = \texttt {tag}(\mathbf {X'}_2 * \mathbf {X'}_3) \wedge \texttt {index}(\mathbf {X}_1) = \texttt {index}(\mathbf {X'}_2 * \mathbf {X'}_3))\\ \end{array}\right) \;\; \wedge \\ (\lnot \mathbf {Z} \wedge \! \lnot \mathbf {Z'}) \Rightarrow \\ \;\;\left( \begin{array}{l} \texttt {sline}(\mathbf {X}_2\! *\! \mathbf {X}_3) = \texttt {sline}(\mathbf {X'}_2\! *\! \mathbf {X'}_3)\; \wedge \\ \texttt {sline}(\mathbf {X}_2\! *\! \mathbf {X}_3) \Rightarrow (\texttt {tag}(\mathbf {X}_2\! *\! \mathbf {X}_3) = \texttt {tag}(\mathbf {X'}_2\! *\! \mathbf {X'}_3) \wedge \texttt {index}(\mathbf {X}_2\! *\! \mathbf {X}_3) = \texttt {index}(\mathbf {X'}_2\! *\! \mathbf {X'}_3)) \end{array}\right) \end{array} \end{aligned}$$

**Fig. 6.**
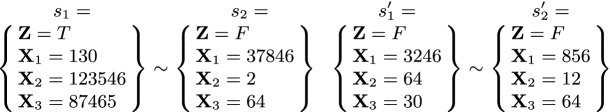
Example test cases when the first 10 cache sets are shared.

We recall that Raspberry Pi 3 has 128 cache sets and 64 bytes per line. Figure 6 shows two pairs of states that satisfy the relation, assuming only the first 10 cache sets are shared. States $s_1$ and $s_2$ lead the program to access the third cache set, while $s'_1$ and $s'_2$ lead the program to access cache sets that are not shared, therefore they generate no observations.

## Test-Case Generation

A test case for a program *P* is a pair of initial states $s_1$, $s_2$ such that *P* produces the same observations when executed from either state, i.e., $s_1 \sim s_2$. The relation as described in Sect. 4 characterizes the space of observationally equivalent states, so a simple but naive approach to test-case generation consists in querying the SMT solver for a model of this relation. The model that results from the query gives us two concrete observationally equivalent values for the registers that affect the observations of the program, so at this point we could forward these to our testing infrastructure to perform the experiment on the hardware.

However, the size of an observational equivalence class can be enormous, because there are many variations to the initial states that cannot have effects on the channels available to the attacker. Choosing a satisfying assignment for the entire relation every time without any extra guidance risks producing many test cases that are too similar to each other, and thus unlikely to find counterexamples. For instance, the SMT solver may generate many variations of the test case $(s_1,s_2)$ in Fig. 6 by iterating over all possible values for register $X_2$ of state $s_1$, even if the value of this register is immaterial for the observation.

In practice, we explore the space of observationally equivalent states in a more systematic manner. To this end, Scam-V supports two mechanisms to guide the selection of test cases: *path enumeration* and *term enumeration*. Path enumeration partitions the space according to the combination of symbolic execution paths that are taken, whereas term enumeration partitions the space according to the value of a user-supplied BIR expression. In both cases, the partitions are explored in round-robin fashion, choosing one test case from each partition in turn. To make the queries to the SMT solver more efficient, we only generate a fragment of the relation that corresponds to the partition under test.

**Path Enumeration.** Every time we have to generate a test case, we first select a pair $(\sigma _1, \sigma _2) \in \Sigma \times \Sigma$ of symbolic states as per Sect. 4, which identifies a pair of paths $(\mathbf {p}_{\sigma _1}, \mathbf {p}_{\sigma _2})$. The chosen paths vary in each iteration in order to achieve full path coverage. The query given to the SMT solver then becomes^5^

$$ \mathbf {p}_{\sigma _1}(s_1) \wedge \mathbf {p}_{\sigma _2}(s_2) \wedge \mathbf {l}_{\sigma _1}(s_1) = \mathbf {l}_{\sigma _2}(s_2) $$

Since the meat of the relation is a conjunction of implications, this is a natural partitioning scheme that ensures all conjuncts are actually explored. Note that without this mechanism, the SMT solver could always choose states that only satisfy one and the same conjunct. To guide this process even further, the user can supply a *path guard*, which is a predicate on the space of paths. Any path not satisfying the guard is skipped, allowing the user to avoid exploring unwanted paths. For example, for the program in Fig. 3 we can use a path guard to force the test generation to select only paths that produce no observations: e.g., $(\mathbf {Z} \Rightarrow \lnot \texttt {sline}(\mathbf {X}_1)) \wedge (\lnot \mathbf {Z} \Rightarrow \lnot \texttt {sline}(\mathbf {X}_2 * \mathbf {X}_3))$.

**Term Enumeration.** In addition to path enumeration, we can choose a BIR expression *e* that depends on the symbolic state, and a range *R* of values to enumerate. Every query also includes the conjuncts $e_{\sigma _1} = v_1 \wedge e_{\sigma _2} = v_2$ where $v_1,v_2\in R$ and such that the $v_i$ are chosen to achieve full coverage of $R \times R$. Term enumeration can be useful to introduce domain-specific partitions, provided that $R\times R$ is small enough. For example, this mechanism can be used to ensure that we explore addresses that cover all possible cache sets, if we set *e* to be a mask that extracts the cache set index bits of the address. For example, for the program in Fig. 3 we can use $\mathbf {Z} * \texttt {index}(\mathbf {X}_1) + (1 - \mathbf {Z}) * \texttt {index}(\mathbf {X}_2 * \mathbf {X}_3)$ to enumerate all combinations of accessed cache sets while respecting the paths.

## Implementation

The implementation^6^ of Scam-V is done in the HOL4 theorem prover using its meta-language, i.e., SML. Scam-V relies on the binary analysis platform HolBA for transpiling the binary code of test programs to the BIR representation. This transpilation uses the existing HOL4 model of the ARMv8 architecture [16] for giving semantics to ARM programs. In order to validate the observational models of Sect. 2.3, we extended the transpilation process to inline observation statements into the resulting BIR program. These observations represent the observational power of the side channel. In order to compute possible execution paths of test programs and their corresponding observations, which are needed to synthesize the observational equivalence relation of Sect. 4, we implemented a symbolic execution engine in HOL4. All program generators from Sect. 3 as well as the weakest relation synthesis from Sect. 4 and the test-case generator from Sect. 5 are implemented as SML libraries in Scam-V. The latter uses the SMT solver Z3 [14] to generate test inputs. For conducting the experiments in this paper, we used Raspberry Pi 3 boards equipped with ARM Cortex-A53 processors implementing the ARMv8-A architecture.

**Fig. 7.**
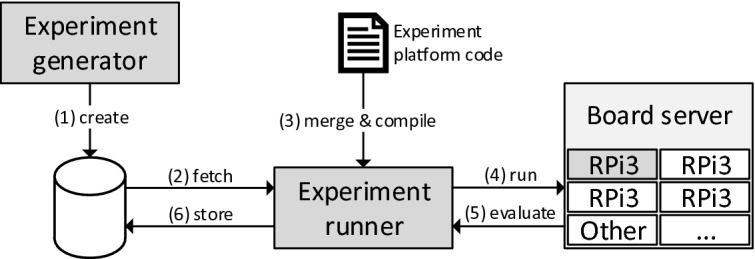
Experiment handling design with numbered steps. This showcases the workflow for producing, preparing, executing and evaluating one experiment.

The Scam-V pipeline generates programs and pairs of observationally equivalent initial states (test cases) for each program. Each combination of a program with one of its test cases is called an *experiment*. After generating experiments, we execute them on the processor implementation of interest to examine their effects on the side channel. Figure 7 depicts the life of a single experiment as goes through our experiment handling design. This consists of: (step 1) generating an experiment and storing it in a database, (step 2) retrieving the experiment from the database, (step 3) integrating it with *experiment-platform code* and compiling it into platform-compatible machine code, and (step 4–6) executing the generated binary on the real board, as well as finally receiving and storing the experiment result.

The experiment-platform code configures page tables to setup *cacheable* and *uncacheable* memory, clears the cache before every execution of the program, and inserts memory barriers around the experiment code. The platform executes in ARM TrustZone, which enables us to use privileged debug instructions to obtain the cache state directly for comparison after experiment execution.

The way in which we compare final cache states for distinguishability depends on the attacker and observational model in question. For multi-way cache, we say two states are *indistinguishable* if and only if for each valid entry in one state, there is a valid entry with the same cache tag in the corresponding cache set of the other state and vice versa. For the partitioned multi-way cache, we check the states in the same way, except we do it only for a subset of the cache sets (see Sect. 7.2 for details on the exact partition). For the direct-mapped cache, we compare how many valid cache lines there are in each set, disregarding the cache tags. These comparison functions have been chosen to match the attacker power of the relaxed models in Definitions 6, 7, and 8 respectively.

## Results

Since the ARM-v8 experimentation platform runs as bare-metal code, there are no background processes or interrupts. Despite this fact, our measurements may contain noise due to other hardware components that share the same memory subsystem, such as the GPU, and because our experiments are not synchronized with the memory controller. In order to simplify repeatability of our experiments, we execute each experiment 10 times and check for discrepancies in the final state of the data cache. Unless all executions give the same result, this experiment is classified as *inconclusive* and excluded from further analysis.

### Direct-Mapped Cache Observational Model

First, we want to make sure that Scam-V can invalidate unsound observational models in general. For this purpose, we generated experiments that use the model of Definition 8, i.e., for every memory access in BIR we observe the cache set index of the address of the access. We know that this is not a sound model for Raspberry Pi 3, because the platform uses a 4-way cache. Table 1.1 shows that both the random program generator and the monadic load generator uncovered counterexamples that invalidated this observational model.

### Partitioned Cache Observational Model

Next, we consider the partitioned cache observational model from Definition 7. That is, we partition the L1 cache of the Raspberry Pi 3 into two contiguous regions and assume that the attacker has only access to the second region. Due to the prefetcher of Cortex-A53 we expect this model to be unsound and indeed we could invalidate it.

To this end, we generated experiments for two variations of the model. Variation A splits the cache at cache set 61, meaning that only cache sets 61–127 were considered accessible to the attacker. Variation B splits the cache at cache set 64 (the midpoint), such that cache sets 64–127 were considered visible. The following program is one of the counterexamples for variation A that have been discovered by Scam-V using the monadic program generator.
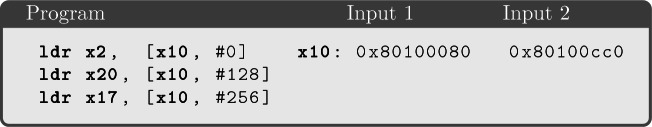

**Table 1.** Invalidation of cache and faulty observational models.

(1.1)	Observations	Cache set index only (Definition 8)		
	Programs	Monadic load generator	Random program generator	
	Experiments	39660	20872	
	- Inconclusive	0	1	
	- Counterexample	19	18	
(1.2)	Experiment set	Variation A	Variation B	
	Observations	Page unaligned cache partitioning (Definition 7)	Page aligned cache partitioning (Definition 7)	
	Programs	Monadic stride generator		
	Experiments	36160	37843	
	- Inconclusive	5426	6967	
	- Counterexample	3460	0	
(1.3)	Observations	Cache tag and set index (Definition 6)		
	Programs	Random program generator	Monadic generator	
			Loads	Previction
	Experiments	20256	23120	23290
	- Inconclusive	2	0	0
	- Counterexample	0	5	16
(1.4)	Observations	Cache tag and set index (Definition 6)		
	Programs	Random program generator		
	Experiments	22321		
	- Inconclusive	0		
	- Failure	308		

The counterexample exploits the fact that prefetching fills more lines than those loaded by the program, provided the memory accesses happen in a certain stride pattern. Thus, it essentially needs to have two properties: (i) two different starting addresses for the stride, $a_1$ and $a_2$, with a cache set index that is lower than 61 to avoid any observations in the model, and thus satisfying observational equivalence, and (ii) one of $a_1$ and $a_2$ is close enough to the partition boundary. In this case, automatic prefetching will continue to fill lines in subsequent sets, effectively crossing the boundary into the attacker-visible region.

In our experiments, we used a path guard to generate only states that produce only memory accesses to the region of the cache that is not visible by the attacker. Additionally, we used term enumeration to force successive test cases to start a stride on a different cache set and therefore cover the different cache set indices. Without this guidance, the tool could generate only experiments that affect the lower sets of the cache and never explore scenarios that affect the sets with indices closer to the split boundary.

For variation B, we have not found such a counterexample. The only difference is that the partition boundary is on line 64, which means that each partition fits exactly in a small page (4K). We conjecture that the prefetcher does not perform line fills across small page (4K) boundaries. This could be for performance reasons, as crossing a page boundary can involve a costly page walk if the next page is not in the TLB. If this is the case, it would seem that it is safe to use prefetching with a partitioned cache, provided the partitions are page-aligned. Table 1.2 summarizes our experiments for this model.

### Multi-way Cache Observational Model

In the remaining experiments, we consider the model of Definition 6 and we assume that the attacker has access to the complete L1 cache. Even if we expected this model to be sound, our experiments (Table 1.3) identified several counterexamples. We comment on two classes of counterexamples below.

**Previction.** Some counterexamples are due to an undocumented behavior that we called “previction” because it causes a cache line to be evicted before the corresponding cache set is full. The following program compares  and  and executes a sequence of three loads. In case of equality, fourteen  are executed between the first two loads.
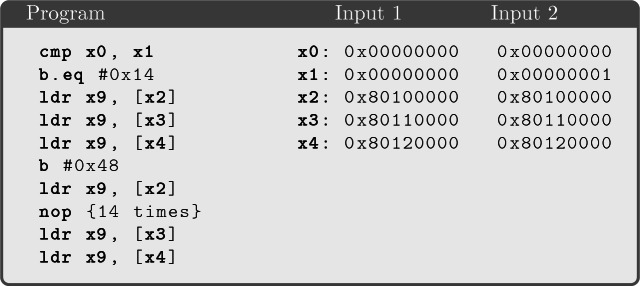


 and  are two states that exercise the two execution paths and have the same values for  ,  and  , hence the two states are observationally equivalent. Notice that all memory loads access cache set 0. Since the cache is 4-way associative and the cache is initially empty, we expect no eviction to occur.

Executions starting in  behave as expected and terminate with the addresses of  ,  , and  in the final cache state. However, the execution from  leads to a previction, which causes the final cache state to only contain the addresses of  and  . The address of  has been evicted even if the cache set is not full. Therefore the two states are distinguishable by the attacker. Our hypothesis is that the processor detects a short sequence of loads to the same cache set and anticipates more loads to the same cache set with no reuse of previously loaded values. It evicts the valid cache line in order to make space for more colliding lines. We note that these cache entries are not dirty and thus eviction is most likely a cheap operation. The execution of a  sequence probably ensures that the first cache line fill is completed before the other addresses are accessed.

**Offset-Dependent Behaviors.** Our experiments identified further counterexamples that invalidate the observational model. In particular, the following counterexample also invalidates the observational model of Definition 5, where cache line offsets are not observable. 
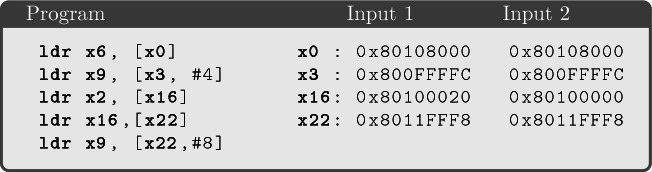
 This program consists of five consecutive load instructions. This program always produces five observations consisting of the cache tag and set index of the five addresses.  and  are observationally equivalent: they only differ for  , which affects the address used for the third load, but the addresses  and  have the same cache tag and set index and only differ for the offset within the same cache line. However, these experiments lead to two distinguishable microarchitectural states. More specifically, execution from  results in the filling of cache set 0, where the addresses of registers  ,  ,  and  are present in the cache, while executions from  leads a cache state where the address of  is not in the cache and has been probably evicted. This effect can be the result of the interaction between cache previction and cache bank collision [9, 40], whose behavior depends on the cache offset. Notice that cache bank collision is undocumented for ARM Cortex-A53. Tromer et al. [46] have shown that such offset-dependent behaviors can make insecure side-channel countermeasures for AES that rely on making accesses to memory blocks (rather than addresses) key-independent.

### Problems in Model Implementations

Additionally to microarchitectural features that invalidate the formal models, our experiments identified bugs of the implementation of the models: (1) the formalization of the ARMv8 instruction set used by the transpiler and (2) the module that inserts BIR observation statements into the transpiled binary to capture the observations that can be made according to a given observational model. Table 1.4 reports problems identified by the random program generator. Some of these failing experiments result in distinguishable states while others result in run-time exceptions. In fact, if the model predicts wrong memory accesses for a program then our framework can generate test inputs that cause accesses to unmapped memory regions. The example program in Fig. 4 exhibits both problems when executed with appropriate inputs.

**Missing Observations.** The second step of our framework translates binary programs to BIR and adds observations to reflect the observational model under validation. In order to generate observations that correspond to memory loads, we syntactically analyze the right-hand side of BIR assignments. For instance, for line *l*2 in Fig. 3 we generate an observation that depends on variable  because the expression of assignment is  . This approach is problematic when a memory load is immaterial for the result of an instruction. For example,  and  instructions load from memory to a register that is constantly zero. The following program loads from  into  . 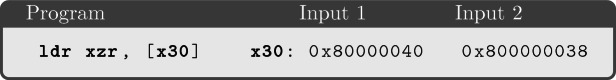 The translation of this instruction is simply  : there is no assignment that loads from  because the register  remains zero. Therefore, our model generates no observations and any two input states are observationally equivalent. The ARM specification does not clarify that the microarchitecture can skip the immaterial memory load. Our experiments show that this is not the case and therefore our implementation of the model is not correct. In fact, the program accesses cache set $\texttt {index}(0x80000040) = 1$ for  and cache set $\texttt {index}(0x80000038) = 0$ for  , which results in distinguishable states. Moreover, by not taking into account the memory access our framework generates some tests that set  to unmapped addresses and cause run-time exceptions.

**Flaw in HOL4 ARMv8 ISA Model.** Our tool has identified a bug of the HOL4 ARMv8 ISA model. This model has been used in several projects [8, 17] as the basis for formal analysis and is used by our framework to transform ARM programs to BIR programs. Despite its wide adoption, we identified a problem in the semantics of instructions *Compare and Branch on Zero* (CBZ) and *Compare and Branch on Non-Zero* (CBNZ). These instructions implement a conditional jump based on the comparison of the input register with zero. While CBZ jumps in case of equality, CBNZ jumps in case of inequality. However, our tests identified that CBNZ wrongly behaves as CBZ in the HOL4 model.

## Related Work

**Hardware Models.** Verification approaches that take into account the underlying hardware architecture have to rely on a formal model of that architecture. Commercial instruction set architectures (ISAs) are usually specified mostly in natural language, and their formalization is an active research direction. For example, Goel et al. [24] formalize the ISA of x86 in ACL2, Morrisett et al. [37] model the x86 architecture in the Coq theorem prover, and Sarkar et al. [42] provide a formal semantics of the x86 multiprocessor ISA in HOL. Moreover, domain-specific languages for ISAs have been developed, such as the L3 language [19], which has been used to model the ARMv7 architecture. As another example, Siewiorek et al. [44] proposed the *Instruction-Set Processor* language for formalizing the semantics of the instructions of a processor.

**Processor Verification and Validation.** To gain confidence in the correctness of a processor model, it needs to be verified or validated against the actual hardware. This problem has received considerable attention lately. There are white-box approaches such as the formal verification that a processor model matches a hardware design [10, 18]. These approaches differ from ours in that they try to give a formal guarantee that a processor model is a valid abstraction of the actual hardware, and to achieve that they require the hardware to be accessible as a white box. More similar to ours are black-box approaches that validate an abstract model by randomly generated instructions or based on dynamic instrumentation [20, 29]. Combinations of formal verification and testing approaches for hardware verification and validation have also been considered [11].

In contrast to our work, all of the approaches above are limited to functional correctness, and validation is limited to single-instruction test cases, which we show to be insufficient for information flow properties. Going beyond these restrictions is the work of Campbell and Stark [12], who generate sequences of instructions as test cases, and go beyond functional correctness by including timing properties. Still, neither their models nor their approach is suitable to identify violations of information flow properties.

**Validating Information Flow Properties.** To the best of our knowledge, we present the first automated approach to validate processor models with respect to information flow properties. To this end, we build on the seminal works of McLean [35] on non-interference, Roscoe [41] on observational determinism, and Barthe et al. [7] on self-composition as a method for proving information flow properties. Most closely related is the work by Balliu et al. [6] on *relational analysis* based on *observational determinism*.

These approaches are based on the different observational models that have been proposed in the literature. For example, the program counter security model [36] has been used when the execution time depends on the control flow of the victim. Extensions of this model also make observable data that can affect execution time of an instruction, or memory addresses accessed by the program to model timing differences due to caching [4].

Many analysis tools use these observational models. Ct-verif [3] implements a sound information flow analysis by proving observational equivalence constructing a product program. CacheAudit [15] quantifies information leakage by using abstract interpretation.

The risks of using unsound models for such analyses have been demonstrated by the recent Spectre attack family [32], which exploits speculation to leak data through caches. Several other architectural details require special caution when using abstract models, as some properties assumed by the models could be unmet. For instance, cache clean operations do not always clean residual state in implementations of replacement policies [21]. Furthermore, many processors do not provide sufficient means to close all leakage, e.g., shared state cannot be cleaned properly on a context switch [22]. Finally, it has been shown that fixes relying on too specific assumptions can be circumvented by modifying the attack [43], and that attacks are possible even against formally verified software if the underlying processor model is unsound [28]. For these reasons, validation of formal models by directly measuring the hardware is of great importance.

## Concluding Remarks

We presented Scam-V, a framework for automatic validation of observational models of side channels. Scam-V uses a novel combination of symbolic execution, relational analysis, and observational models to generate experiments. We evaluated Scam-V on the ARM Cortex-A53 processor and we invalidated all models of Sect. 2.3, i.e., those with observations that are cache-line-offset-independent.

Our results are summarized as follows: (i) in case of cache partitioning, the attacker can discover victim accesses to the other cache partitions due to the automatic data prefetcher; (ii) the Cortex-A53 prefetcher seems to respect 4K page boundaries, like in some Intel processors; (iii) a mechanism of Cortex-A53, which we called previction, can leak the time between accesses to the same cache set; (iv) the cache state is affected by the cache line offset of the accesses, probably due to undocumented cache bank collisions like in some AMD processors; (v) the formal ARMv8 model had a flaw in the implementation of CBNZ; (vi) our implementation of the observational model had a flaw in case of loads into the constant zero register. Moreover, since the microarchitectural features that lead to these findings are also available on other ARMv8 cores, including some that are affected by Spectre (e.g. Cortex A57), it is likely that similar behaviors can be observed on these cores, and that more powerful observational models, including those that take into account Spectre-like effects, may also be unsound.

These promising results show that Scam-V can support the identification of undocumented and security-relevant features of processors (like results (ii), (iii), and (iv)) and discover problems in the formal models (like results (v) and (vi)). In addition, users can drive test-case generation to conveniently explore classes of programs that they suspect would lead to side-channel leakage (like in result (i)). This process is enabled by path and term enumeration techniques as well as custom program generators. Moreover, Scam-V can aid vendors to validate implementations with respect to desired side-channel specifications.

Given the lack of vendor communication regarding security-relevant processor features, validation of abstract side-channel models is of critical importance. As a future direction of work, we are planning to extend Scam-V for other architectures (e.g. ARM Cortex-M0 based microcontrollers), noisy side channels (e.g. time and power consumption), and other side channels (e.g. cache replacement state). Moreover, we are investigating approaches to automatically repair an unsound observational model starting from the counterexamples, e.g., by adding state observations. Finally, the theory in Sect. 4 can be used to develop a certifying tool for verifying observational determinism.

## Footnotes

1 Terminating programs which do not cause run-time exceptions and emit observations required by the analysis.

2 https://hol-theorem-prover.org.

3 https://www.raspberrypi.org.

4 We consider only terminating programs.

5 Note that this is equivalent to taking a fragment of the observational equivalence relation, specifically the case when $\mathbf {p}_{\sigma _1}(s_1) \wedge \mathbf {p}_{\sigma _2}(s_2)$ holds.

6 Our implementation of Scam-V is embedded in HolBA, which is available at https://github.com/kth-step/HolBA. Our extendable experimentation platform consists of several “EmbExp-*” repositories available at https://github.com/kth-step.
